# 4,6′-Anhydrooxysporidinone from *Fusarium lateritium* SSF2 Induces Autophagic and Apoptosis Cell Death in MCF-7 Breast Cancer Cells

**DOI:** 10.3390/biom11060869

**Published:** 2021-06-11

**Authors:** Dahae Lee, Sanghee Shim, Kisung Kang

**Affiliations:** 1 College of Korean Medicine, Gachon University, Seongnam 13120, Korea; pjsldh@gachon.ac.kr; 2 Natural Products Research Institute, College of Pharmacy, Seoul National University, Seoul 08826, Korea; sanghee_shim@snu.ac.kr

**Keywords:** *Fusarium lateritium* SSF2, autophagy, apoptosis, MCF-7

## Abstract

Previous studies have reported that 4,6′-Anhydrooxysporidinone (SSF2-2), isolated from *Fusarium lateritium* SSF2, has neuroprotective effects on the HT-22 hippocampal neuronal cell line. However, the anti-cancer effect of SSF2-2 remains unclear. Here, we examined the viability of MCF-7 human breast cancer cells treated with SSF2-2 or left untreated using a cell viability assay kit. The underlying molecular mechanism was further investigated by Western blotting and immunocytochemistry studies. The results demonstrated that SSF2-2 inhibited the viability of MCF-7 cells. Treatment with SSF2-2 increased the levels of cleaved caspase-9, cleaved caspase-7, poly (ADP-ribose) polymerase (PARP), and LC3B. Additionally, SSF2-2 significantly increased the conversion of LC3-I to LC3II and LC3-positive puncta in MCF-7 cells.

## 1. Introduction

Breast cancer is one of the leading causes of death among women worldwide [1]. It is the most common type of malignant tumor that can be treated with chemotherapy, radiotherapy, and surgery [2]. However, these treatments have undesirable side effects, as they not only affect breast cancer cells but have undesirable effects on normal cells as well. The identification of effective novel therapeutic strategies remains a necessity in the treatment of breast cancer [3]. We have continually studied the effects of natural products on the apoptotic cell death of breast cancer cells [4,5,6,7,8,9]. Accumulating studies on the identification of anticancer substances from natural products have reported that apoptosis and autophagy are important pathways in the death of cancer cells [10].

Endophytic fungi are a source of compounds with unique chemical skeletons and possess interesting biological activities. Sclerotiorin, isolated from the endophytic fungus *Cephalotheca faveolata*, induces apoptosis in HCT-116 human colon cancer cells [11]. A study reported that dicatenarin, isolated from the endophytic fungus *Penicillium pinophilum*, induces apoptosis in MIA PaCa-2 human pancreatic cancer cells [12]. Another study demonstrated that xylarione A, isolated from the endophytic fungus *Xylaria psidii,* induces apoptosis in MIA PaCa-2 cells [13]. The anticancer activity of cytochalasin H, isolated from the endophytic fungus *Phomopsis liquidambari*, is reported to be mediated via the inhibition of cell viability and apoptosis in A549 human lung cancer cells [14]. Additionally, the anticancer effects of chaetocochins G, isolated from an endophytic fungus, *Chaetomium* sp. 88194, and orsellinic acid esters, isolated from the endophytic fungus *Ch. globosum*, on MCF-7 human breast cancer cells have also been investigated [15,16]. However, the detailed mechanism underlying their anticancer effects on MCF-7 cells remains unclear.

In our previous study, we determined that 4,6′-anhydrooxysporidinone (SSF2-2), isolated from the endophytic fungus *Fusarium lateritium* SSF2, has a neuroprotective effect in the HT-22 hippocampal neuronal cell line. The effect is mediated via the inhibition of glutamate-induced oxidative stress and apoptosis. In this study, low concentrations of 2.5 and 5 μM SSF2-2 were effective [17]. The anticancer effects of compounds isolated from *F. lateritium* SSF2 have not been explored to date. In our continuing interest in evaluating the bioactive properties of tricyclic pyridone alkaloids isolated from *F. lateritium* SSF2, we studied the anticancer effect of these tricyclic pyridone alkaloids on MCF-7 cells. We additionally investigated the detailed molecular mechanism underlying the anticancer effect of SSF2-2 by focusing on the apoptotic and autophagic pathways.

## 2. Materials and Methods

### 2.1. Extraction and Isolation

*F. lateritium* SSF2 was cultivated on PDA media on a large scale and subsequently extracted with organic solvents to obtain the MeOH extracts (1.78 g). These extracts were successively extracted with n-hexane, CHCl_3_, and EtOAc to obtain the polarity-dependent fractions. Among these, the CHCl_3_ fraction was selected for chemical investigation and subjected to a series of chromatographic procedures, which yielded three 4-hydroxyl pyridone-type compounds (**1**–**3**). The detailed procedures of separation and purification are described in our earlier report [11].

### 2.2. Cell Culture

MCF-7 cells were purchased from the American Type Culture Collection (Manassas, VA, USA) and cultured in Roswell Park Memorial Institute (RPMI) 1640 medium (Corning, Manassas, VA, USA) supplemented with 10% fetal bovine serum (Atlas, Fort Collins, CO, USA) and 1% penicillin/streptomycin (Invitrogen Co., Grand Island, NY, USA). The MCF-7 cells were grown to 80% confluence at 37 °C in a humidified atmosphere containing 5% CO_2_.

### 2.3. Assessment of Cell Viability

The viability of MCF-7 cells was assessed following treatment with SSF2-1, SSF2-2, and SSF2-3 using an Ez-CyTox assay kit (Daeil Lab Service Co., Seoul, Korea), as described in the literature [18]. Briefly, the MCF-7 cells were seeded onto 96-well plates and incubated for 24 h at 37 °C in a humidified atmosphere containing 5% CO_2_. The cells were subsequently treated with SSF2-1, SSF2-2, and SSF2-3 and incubated for the indicated durations from 0 to 24 h at 37 °C in a humidified atmosphere containing 5% CO_2_. For quantifying the cell viability, the cells that had been incubated for the desired durations were incubated with EZ-CyTox reagents for an additional 30 min at 37 °C in a humidified atmosphere containing 5% CO_2_, and the optical density of each well was determined by measuring the absorbance at 450 nm using a microplate reader (Molecular Device, Palo Alto, CA, USA).

### 2.4. Immunocytochemistry

The MCF7 cells were grown on a multi-well cell culture slide (SPL Life Sciences, Pocheon, South Korea) and allowed to adhere for 24 h. The cells were subsequently treated with 25 and 50 μM SSF2-2. After 12 h of exposure, the cells were fixed for 15 min with a solution of 4% paraformaldehyde. The cells were subsequently permeabilized by incubation with 0.1% Triton X-100 for 5 min and blocked by incubation with 1% bovine serum albumin containing 0.01% Triton X-100 for 1 h at room temperature (RT). After overnight incubation with a primary antibody specific for LC3B at 4 °C, the cells were incubated for 1 h with Alexa Fluor 488-conjugated goat anti-rabbit IgG at RT. The cells were subsequently mounted with mounting medium (Vectashield, Burlingame, CA, USA) containing 4′,6-diamidino-2-phenylindole (DAPI) and observed under a fluorescent microscope (IX71, Olympus, Tokyo, Japan) equipped with a CCD camera (C4742-95, Hamamatsu Photonics, Hamamatsu, Japan) with slight modifications, as previously described [19]. The quantification of mean LC3-stained puncta per nuclei was carried out with the ImageJ software (National Institutes of Health, Bethesda, MD, USA), as previously described [20].

### 2.5. Western Blot Analysis

The MCF-7 cells were plated in 6-well plates at 37 °C in a humidified atmosphere containing 5% CO_2_. For the additional experiment, the 5 mM autophagy inhibitor 3-methyladenine (3-MA) (Sigma, St. Louis, MO, USA) was pre-treated 1 h prior to the SSF2-2 treatment.

After 24 h, the cells were treated with 25 and 50 μM SSF2-2 and incubated for 12 h or 24 h at 37 °C in a humidified atmosphere containing 5% CO_2_. The total protein was extracted from the MCF7 cells using a RIPA buffer (50 mM Tris-HCl, 150 mM NaCl, 1 mM EDTA, 1% sodium deoxycholate, 1% NP40, and 0.1% SDS) containing phosphatase inhibitors (5 mM Na_3_VO_4_, 1 mM NaF) and a protease inhibitor cocktail. The total proteins were separated by SDS polyacrylamide gel electrophoresis and transferred to a polyvinylidene fluoride membrane. The membranes were probed with specific primary antibodies against cleaved caspase-7 (# 9491S, 1:1000), cleaved caspase-9 (# 20750S, 1:1000), poly (ADP-ribose) polymerase (PARP) (# 5625S, 1:1000), LC3B (# 43566S, 1:1000), p53 (# 2527S, 1:1000), and glyceraldehyde 3-phosphate dehydrogenase (GAPDH) (# 2118S, 1:1000). After incubation with the primary antibodies for 1 h, the membranes were incubated with the appropriate secondary antibodies conjugated with horseradish peroxidase for 1 h. All the antibodies were purchased from Cell Signaling Technology (Danvers, MA, USA). The immunoreactive bands were visualized with ECL solution (GE Healthcare, Little Chalfont, UK), using a Fusion Solo imaging system (FUSION Solo, PEQLAB Biotechnologie GmbH, Erlangen, Germany). The immunoreactive bands were analyzed using the ImageJ software (National Institutes of Health, Bethesda, MD, USA) and represented in terms of fold increases compared with those of the control cells, as previously described [21].

### 2.6. Quantitative Analysis for Apoptotic Cells

The MCF-7 cells were plated in 6-well plates at 37 °C in a humidified atmosphere containing 5% CO_2_. After 24 h, the cells were treated with 25 and 50 μM SSF2-2 and incubated for 24 h at 37 °C in a humidified atmosphere containing 5% CO_2_. For determining apoptotic cell death, the cells were stained with 5 μL annexin V and 1 μL propidium iodide (PI) for 30 min at RT using a Tali Image-based Cytometer Apoptosis Kit (Invitrogen, Life Technologies, Carlsbad, CA, USA). The percentage of apoptotic cells was counted in 10 randomly selected fields per slide at a 40× magnification using a Tali Image-Based Cytometer (Invitrogen; Temecula, CA, USA).

### 2.7. Cell Staining with Hoechst 33342

The MCF-7 cells were plated in 6-well plates at 37 °C in a humidified atmosphere containing 5% CO_2_. After 24 h, the cells were treated with 25 and 50 μM SSF2-2 and incubated for 24 h at 37 °C in a humidified atmosphere containing 5% CO_2_. For detecting nuclear condensation, the cells were stained with 2 μL Hoechst 33342 for 10 min at RT. The cells were subsequently observed under a fluorescent microscope (IX71, Olympus, Tokyo, Japan) equipped with a CCD camera (C4742-95, Hamamatsu Photonics, Hamamatsu, Japan).

### 2.8. Statistical Analyses

Statistical significance was determined by an analysis of variance (ANOVA) followed by a multiple comparison test with Bonferroni adjustment. Statistical significance was considered at *p* < 0.05.

## 3. Results

### 3.1. SSF2-2 Inhibits the Viability of MCF-7 Cells

**Figure 1:**
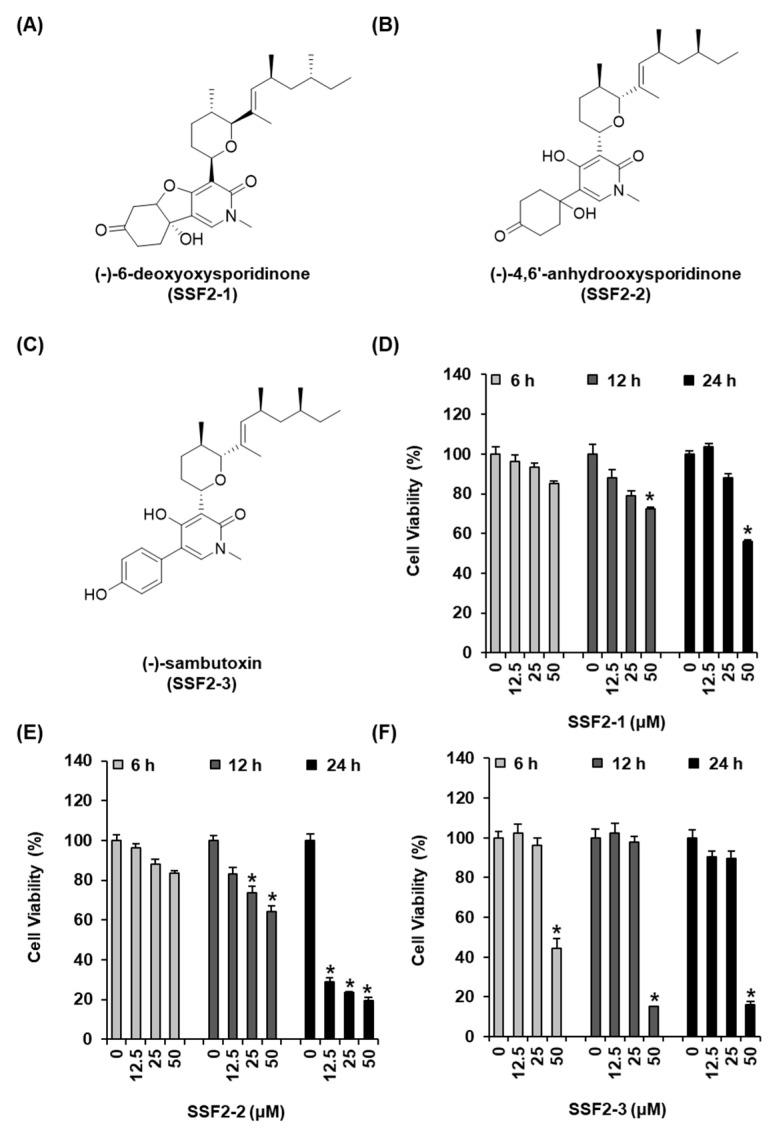
SSF2-1, SSF2-2, and SSF2-3 inhibited the viability of MCF-7 human breast cancer cells. The chemical structures of (**A**) SSF2-1, (**B**) SSF2-2, and (**C**) SSF2-3 were isolated from cultures of *F. lateritium* SSF2. The MCF-7 cells were incubated with various concentrations of (**D**) SSF2-1, (**E**) SSF2-2, and (**F**) SSF2-3 for 6, 12, or 24 h. The 0.5% (*v/v*) dilution of DMSO in RPMI 1640 medium was used as a vehicle control. Cell viability was assessed using the Ez-CyTox reagent. The data represent the mean ± S.E.M., *n* = 3, * *p* < 0.05 compared with the control.

**Figure 2:**
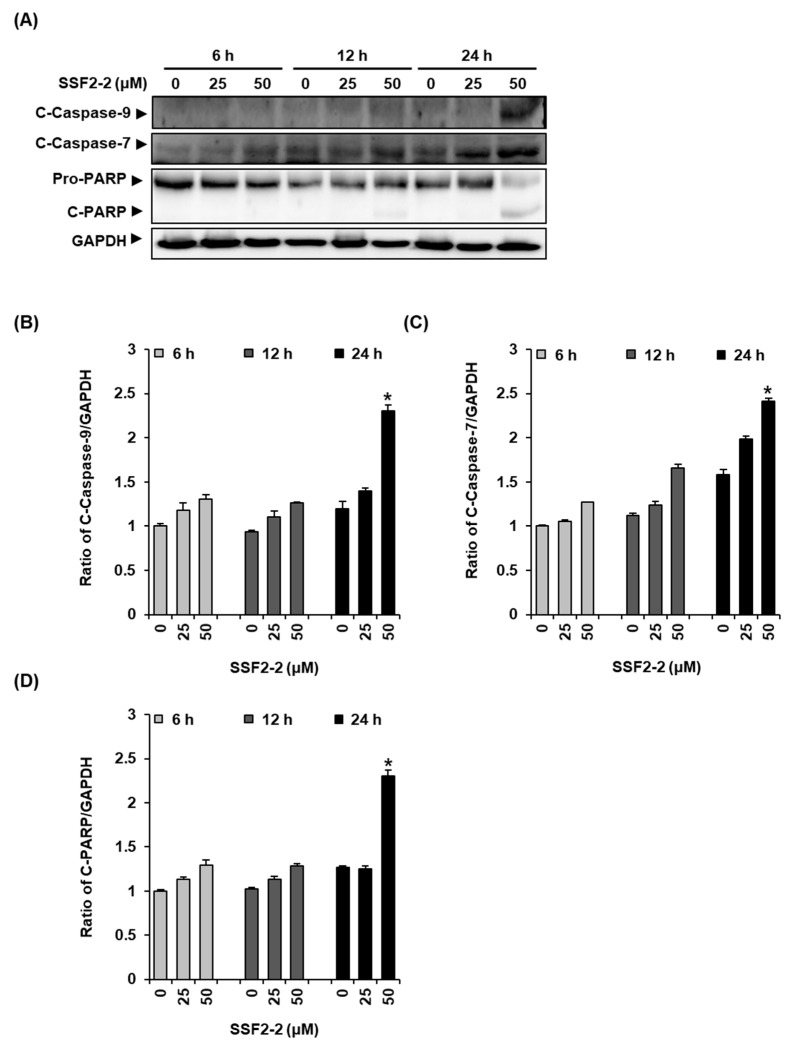
**The** effects of SSF2-2 on the expression of apoptosis-related proteins in MCF-7 human breast cancer cells. (**A**) Representative images of the Western blots for cleaved caspase-9, cleaved caspase-7, PARP, and GAPDH in MCF-7 cells treated with 25 or 50 μM SSF2-2 for 6, 12, or 24 h. (**B**–**D**) Each of the bar graphs present the densitometric quantification of the bands in the Western blots. A 0.5% (*v/v*) dilution of DMSO in RPMI 1640 medium was used as a vehicle control. The data represent the mean ± S.E.M., *n* = 3, * *p* < 0.05 compared with the control.

We first evaluated the effects of SSF2-1, SSF2-2, and SSF2-3, isolated from the *F. lateritium* SSF2 cultures, on the viability of MCF-7 breast cancer cells. The structures of the compounds are depicted in Figure 1A–C. The MCF-7 cells were treated with different concentrations of SSF2-1, SSF2-2, and SSF2-3, ranging from 12.5 to 50 µM, for varying durations of 6, 12, and 24 h. The viability of the MCF-7 cells was inhibited by treatment with SSF2-1 at a concentration of 50 μM. SSF2-1 inhibited the cell viability by 72.51 ± 0.85% and 55.76 ± 1.14% after 12 and 24 h of treatment, respectively (Figure 2D). The inhibitory effect of SSF2-2 on cell viability was both dose- and time-dependent, and the reduction in viability was statistically significant when incubated for 24 h, even when treated with a low dose (12.5 μM) of SSF2-2. After 12 h of treatment with 25 and 50 μM SSF2-2, the viability decreased by 73.68 ± 3.42% and 64.41 ± 2.57%, respectively. Additionally, treatment with SSF2-2 for 24 h strongly inhibited the viability of MCF-7 cells by approximately 29.01 ± 1.88%, 23.57 ± 0.46%, and 19.35 ± 1.89%, at concentrations of 12.5, 25, and 50 μM, respectively (Figure 2E). Treatment with 50 μM of SSF2-3 for 6 h inhibited the cell viability by 44.49 ± 4.92%. Furthermore, treatment with 50 μM of SSF2-3 for 12 and 24 h inhibited the viability of MCF-7 cells by 15.26 ± 0.18% and 15.95 ± 1.81%, respectively (Figure 2F). SSF2-2 was therefore selected for further experimentation in this study.

### 3.2. SSF2-2 Induces Apoptosis in MCF-7 Cells

Western blotting was performed to ascertain whether SSF2-2 increases the levels of proteins involved in the apoptotic pathway in MCF-7 cells. The results demonstrate that the protein levels of cleaved caspase-9 and caspase-7 and cleaved PARP increased after treatment with 50 μM pf SSF2-2 for 24 h in MCF-7 cells (Figure 2A,B).

**Figure 3:**
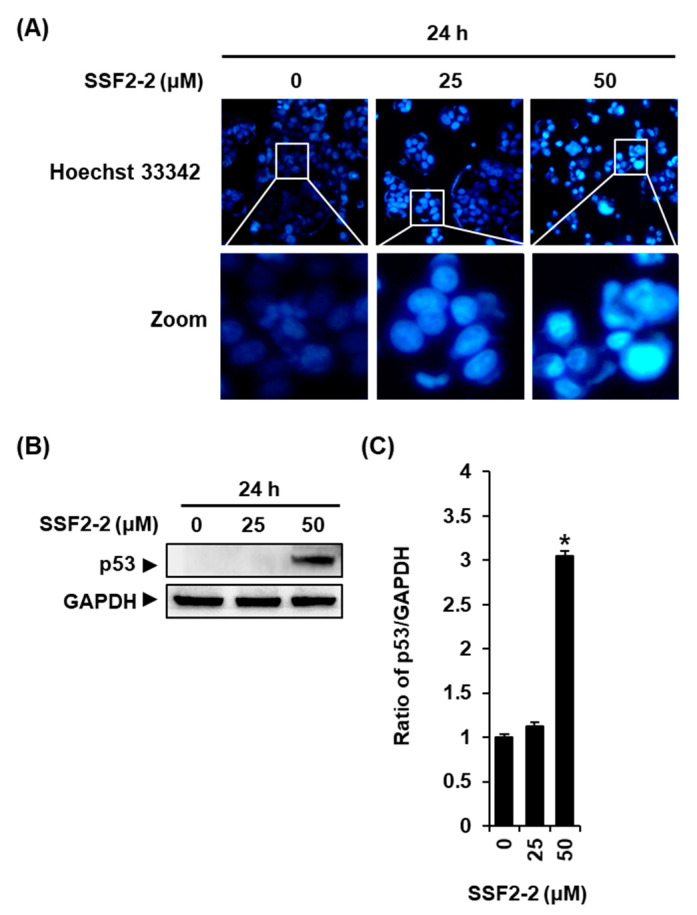
The effects of SSF2-2 on the nuclear morphologies and expression of p53 proteins in MCF-7 human breast cancer cells. (**A**) Representative fluorescence microscopy images of Hoechst 33342 staining in MCF-7 cells treated with 25 or 50 μM SSF2-2 for 24 h (20× magnification). (**B**) Representative images of the Western blots for p53 and GAPDH in MCF-7 cells treated with 25 or 50 μM SSF2-2 for 24 h. (**C**) A bar graph presenting the densitometric quantification of the bands in the Western blots. A 0.5% (*v/v*) dilution of DMSO in RPMI 1640 medium was used as a vehicle control. The data represent the mean ± S.E.M., *n* = 3, * *p* < 0.05 compared with the control.

To stain chromatin DNA, we performed staining with Hoechst 33342 after treatment with 25 or 50 μM SSF2-2. We observed a normal nuclear in vehicle control, while the nucleus became condensed in MCF-7 cells incubated with 50 μM SSF2-2 (Figure 3A). In addition, the protein levels of p53 increased after treatment with 50 μM SSF2-2 for 24 h in MCF-7 cells (Figure 3B,C).

**Figure 4:**
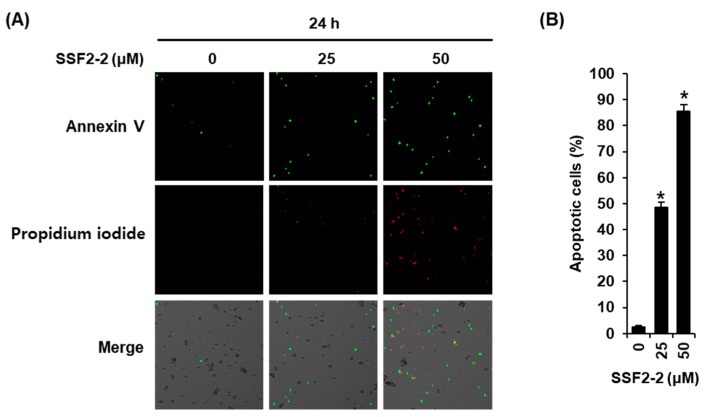
SSF2-2 inhibits the apoptotic cell death of MCF-7 human breast cancer cells. (**A**) Representative images of apoptotic MCF-7 cells stained with annexin V (green fluorescence) and PI (red fluorescence) after treatment with 25 or 50 μM SSF2-2 for 24 h. (**B**) Percentage of annexin V-positive apoptotic cells. The 0.5% (*v/v*) dilution of DMSO in RPMI 1640 medium was used as a vehicle control. A total of 10 different fields (40× magnification) were selected per slide and observed with a Tali Image-Based Cytometer. Representative fields are shown for each condition. The data represent the mean ± S.E.M., *n* = 3, * *p* < 0.05 compared with the control.

In order to further confirm that SF2-2 induces apoptotic cell death in MCF-7 cells, we performed double staining with Annexin V and PI after treatment with 25 or 50 μM SSF2-2. The results demonstrate that the proportion of Annexin V/PI-positive apoptotic cells was higher in the SSF2-2-treated cell population (Figure 4A). The percentage of Annexin V-positive cells significantly increased to 48.57 ± 2.01% and 85.61 ± 2.49% after treatment with 25 or 50 μM SSF2-2, respectively (Figure 4B), indicating that SSF2-2 induces apoptotic cell death in MCF-7 cells.

### 3.3. SSF2-2 Induces Autophgay in MCF-7 Cells

**Figure 5:**
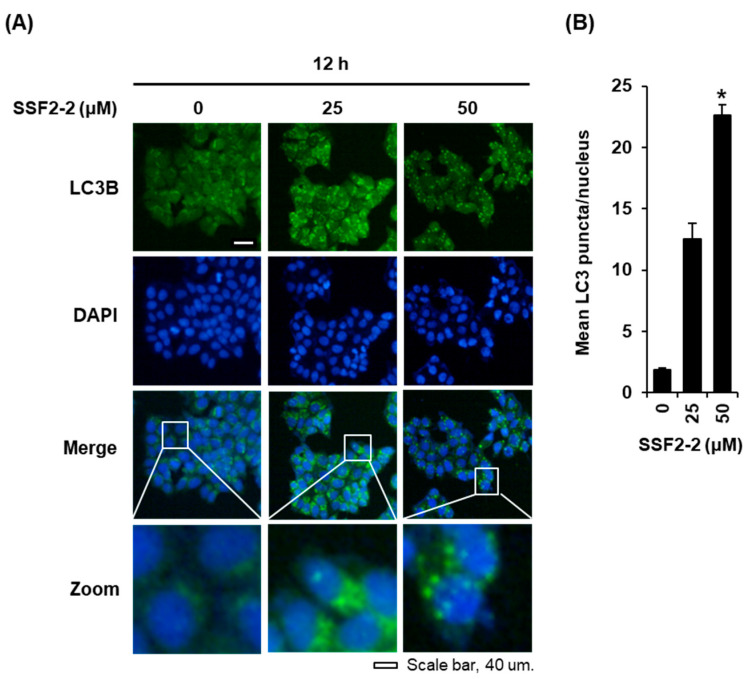
The effect of SSF2-2 on the formation of LC3 puncta in MCF-7 human breast cancer cells. (**A**) Representative fluorescence images of MCF-7 cells stained with anti-LC3B antibody (green) and DAPI (blue) after treatment with 25 or 50 μM SSF2-2 for 12 h. Scale bar = 40 μm in the fluorescent microscope. (**B**) A bar graph presenting the quantification of mean LC3-stained puncta per nuclei. The 0.5% (*v/v*) dilution of DMSO in RPMI 1640 medium was used as a vehicle control. The data represent the mean ± S.E.M., *n* = 3, * *p* < 0.05 compared with the control.

**Figure 6:**
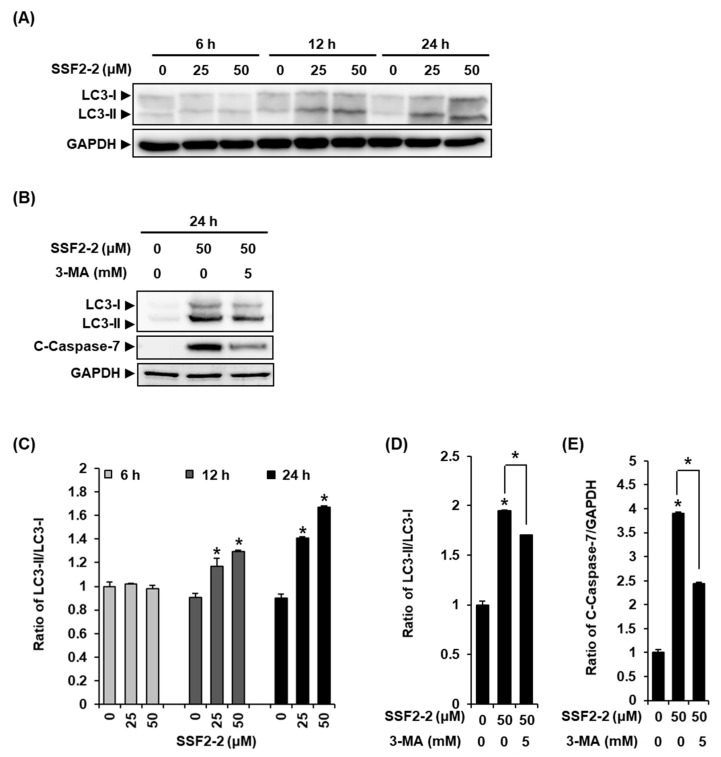
**The** effects of SSF2-2 on the expression of LC3-II in MCF-7 human breast cancer cells. (**A**) Representative images of the Western blots for LC3-II and GAPDH in MCF-7 cells treated with 25 or 50 μM SSF2-2 for 6, 12, or 24 h. (**B**) Representative images of the Western blots for LC3-II, cleaved caspase-7, and GAPDH in MCF-7 cells treated with 50 μM SSF2-2 and 5 mM 3-MA for 24 h. (**C**–**E**) Each bar graph presents the densitometric quantification of the bands observed in the Western blots. The 0.5% (*v/v*) dilution of DMSO in RPMI 1640 medium was used as a vehicle control. The data represent the mean ± S.E.M., *n* = 3, * *p* < 0.05 compared with the control.

We performed the immunofluorescence staining of LC3B in MCF-7 cells treated with 25 or 50 μM SSF2-2 for 12 h to detecting the LC3 puncta. In agreement with the data obtained from Western blotting, we observed a significant increase in the endogenous LC3 puncta by the immunofluorescent staining of MCF-7 cells treated with 25 or 50 μM SSF2-2 for 12 h, indicating that SSF2-2 induced autophagic cell death (Figure 5). To confirm this observation, Western blotting was performed in order to ascertain whether SSF2-2 increases the protein levels of LC3B, the biomarker of autophagy, in MCF-7 cells. The results revealed that the protein levels of LC3B-II increased in MCF-7 cells treated with 25 or 50 μM SSF2-2 for 12 and 24 h (Figure 6A). In addition, the increased protein expression levels of LC3B and cleaved caspase-7 in SSF2-2-incubated MCF-7 cells were decreased when they were co-treated with 3-MA (Figure 6B).

## 4. Discussion

The natural compound, 4,6-anhydrooxysporidinone, has been isolated from the endophytic fungus *F. oxysporum* and the endophytic fungus *F. tricinctum* SYPF 7082. The inhibitory effect of 4,6-anhydrooxysporidinone on the production of nitric oxide and the growth of various cancer cell lines, including the SF-268 human glioma cell line, the A549 and NCI-H460 human lung cancer cell lines, the PANC-1 and MIA Paca-2 human pancreatic cancer cell lines, the PC3 human prostate cancer cell line, and MCF-7 cells have been previously investigated, and the results revealed a weak inhibitory activity [22,23,24].

Here, we report that 4,6-anhydrooxysporidinone, isolated from the endophytic fungus *F. lateritium* SSF2, inhibited the viability of MCF-7 cells. The results of the cell viability assay revealed that 4,6-anhydrooxysporidinone inhibited the viability of MCF-7 cells in a concentration- and time-dependent manner. The detailed molecular mechanism underlying the cytotoxic effect of 4,6-anhydrooxysporidinone in MCF-7 cells was assessed by Western blotting and immunocytochemistry studies, focusing on the apoptotic and autophagic pathways. The activation of caspase-9 and caspase-7 via the mitochondrial pathway is an important mediator of apoptotic cell death [25]. In the mitochondrial apoptotic pathway, the release of cytochrome *c* from the mitochondria results in the formation of the apoptosome, which is followed by the activation of caspase-9, and cleaved caspse-9 triggers the activation of caspase-7 and caspase-3 [26]. It is known that the MCF-7 cells used in the present study are deficient in caspase-3 [27,28,29]. In the later stages of apoptosis, cleaved caspse-7 induces the cleavage of PARP, which prevents energy (ATP and NAD) depletion and the futile repair of DNA double-strand breaks [30]. We therefore investigated whether the protein levels of caspase-9, caspase-7, and PARP would increase after treatment with 4,6-anhydrooxysporidinone. In our study, the protein levels of cleaved caspase-9, cleaved caspase-7, and PARP increased in MCF-7 cells after treatment with 50 μM 4,6-anhydrooxysporidinone for 24 h. These results demonstrated that 50 μM 4,6-anhydrooxysporidinone induced apoptotic cell death via the activation of caspase-9, caspase-7, and PARP, which play key roles in the mitochondrial apoptotic pathway. In addition, the DNA damage of the MCF-7 cells was indicated by Hoechst 33342 staining after treatment with 50 μM 4,6-anhydrooxysporidinone for 24 h. Additionally, the protein levels of P53 increased in MCF-7 cells after treatment with 50 μM 4,6-anhydrooxysporidinone for 24 h. It is known that p53 is activated after DNA damage, and it is responsible for both apoptosis and autophagy [31]. Programmed cell death is not limited to the apoptotic pathway, but may be triggered by other pathways as well. The other pathway of programmed cell death is autophagic cell death. Autophagy acts as a protective mechanism that removes damaged cellular constituents, and its hyperactivation can contribute to cell death [32]. Autophagic cell death is distinguished from apoptotic cell death by the presence of autophagosomes. The conversion of LC3 (LC3B-I to LC3B-II) is necessary for the formation of autophagosomes [33]. Compared to apoptotic cell death, little is known about autophagic cell death; however, autophagy is known to be induced before apoptosis in dying cells [34]. In the present study, 50 μM 4,6′-anhydrooxysporidinone induced apoptotic cell death at 24 h, whereas autophagic cell death was induced at 12 h. The results demonstrate that 25 and 50 μM 4,6′-anhydrooxysporidinone increases the protein levels of LC3B, a biomarker of autophagy, after 12 and 24 h of treatment. The results of immunofluorescence staining reveal a significant increase in endogenous LC3 puncta. We also found that the increased protein expression levels of cleaved caspase-7 in 4,6′-anhydrooxysporidinone-incubated MCF-7 cells were decreased when co-treated with autophagy inhibitor 3-MA, which indicated that 4,6′-anhydrooxysporidinone-induced apoptosis was autophagy-dependent. Collectively, these results indicate that 4,6-anhydrooxysporidinone partially inhibited the viability of MCF-7 cells via apoptotic and autophagic pathways of cell death. Autophagy is known to act independently in parallel pathways of apoptosis or to influence as upstream in the apoptosis pathway [31]. Given the complex link between autophagy and apoptosis pathways, further research is still needed to comprehensively elucidate the anticancer effect of 4,6′-anhydrooxysporidinone and its effects in animal models.

## 5. Conclusions

Taken together, our results demonstrated that 4,6-anhydrooxysporidinone, isolated from the endophytic fungus *F. lateritium*, inhibited the viability of MCF-7 human breast cancer cells by inducing apoptosis and autophagy. The results demonstrated that 4,6-anhydrooxysporidinone induced apoptotic cell death via the activation of the caspase-9, caspase-7, PARP, and p53, which play key roles in the mitochondrial apoptotic pathway. The results further revealed that 4,6-anhydrooxysporidinone increased the protein expression of LC3-II and the endogenous LC3 puncta. Further studies are necessary to elucidate the detailed molecular mechanisms underlying the apoptosis and autophagy induced by 4,6-anhydrooxysporidinone in MCF-7 cells and its effects in experimental animal models.
